# Evaluation of Antimicrobial Durability and Anti-Biofilm Effects in Urinary Catheters Against *Enterococcus faecalis* Clinical Isolates and Reference Strains

**DOI:** 10.4274/balkanmedj.2016.1853

**Published:** 2017-12-01

**Authors:** Didem Kart, Ayşe Semra Kustimur, Meral Sağıroğlu, Ayşe Kalkancı

**Affiliations:** 1 Department of Pharmaceutical Microbiology, Hacettepe University Faculty of Pharmacy, Ankara, Turkey; 2 Department of Medical Microbiology, Gazi University School of Medicine, Ankara, Turkey

**Keywords:** Biofilm, Enterococcus faecalis, gelatinase, urinary catheter, nitrofurazone, silver

## Abstract

**Background:**

*Enterococcus faecalis, Escherichia coli, Staphylococcus epidermidis, Pseudomonas aeruginosa* and *Candida albicans* biofilms are major causes of catheter-associated urinary tract infections. Antimicrobial-coated or impregnated urinary catheters are seen as a possible way to prevent these infections.

**Aims:**

To determine the biofilm-forming ability of 89 *E. faecalis* isolates from urinary tract infections and to compare several urinary catheters for antimicrobial durability and the inhibitory effects on biofilm formation of different laboratory strains and clinical isolates of *E. faecalis*.

**Study Design:**

In vitro experimental study.

**Methods:**

The biofilm forming ability of *E. faecalis* isolates was determined by the crystal violet staining and plate counting methods. For comparison of urinary catheters, biofilms of 45 *E. faecalis* isolates from the catheter samples of hospitalized patients and five laboratory strains of E. coli ATCC25922, S. epidermidis ATCC35984, *P. aeruginosa* ATCC27853, *E. faecalis* ATCC29212 and *C. albicans* ATCC90028 were formed on the catheters in 24-well tissue culture plates. Scanning electron microscopy analysis was performed to observe biofilms.

**Results:**

All 89 *E. faecalis* isolates were found to be biofilm positive. Nitrofurazone-impregnated catheters significantly reduced the cell counts of *E. faecalis* isolates and completely inhibited the formation of *P. aeruginosa* and S. epidermidis biofilms compared with the others. Regarding reduction of biofilm cell counts, a hydrophilic-coated catheter was more effective against *P. aeruginosa*, whereas a silver-coated catheter was found to be more effective against S. epidermidis. The nitrofurazone-impregnated catheter had the best antimicrobial durability.

**Conclusion:**

Urine isolates of *E. faecalis* had considerable ability with respect to biofilm formation. The nitrofurazone-impregnated catheter was the most effective against all tested bacteria; however, the effect of a hydrophilic or silver-coated catheter depends on the species present in it.

Catheter-associated urinary tract infections (CAUTIs) account for 80% of all hospital-acquired infections and correlate with the presence of microbial biofilms that cause catheter blockage (1). Bacterial biofilms are defined as communities of sessile bacteria which adhere to either biotic or abiotic surfaces and are encased in an extracellular matrix (ECM). One of the well-known properties of biofilm cells is that they display resistance to antimicrobial agents at concentrations up to a thousand times higher than those used to destroy their planktonic forms (2,3). It has been estimated that 65%-80% of infections occurring in the human body are biofilm mediated (3).

In the CAUTI guidelines, the utilization of antimicrobial-coated or impregnated urinary catheters is suggested as a possible preventive strategy to fight these infections (4,5). Silver alloy and nitrofurazone are the most frequently used antimicrobials in urinary catheters. It has been shown that silver alloy- and nitrofurazone-impregnated catheters significantly reduce the incidence of asymptomatic bacteriuria in hospitalized patients after less than 1 week of catheterization (6). Another strategy to inhibit microbial attachment to the catheter surface is a hydrogel coating to reduce urethral friction (7).

A variety of species such as Escherichia coli, Staphylococcus epidermidis, Pseudomonas aeruginosa and Candida albicans are considered significant causative agents of nosocomial urinary tract infections (UTIs). Enterococcus species have also been associated with various nosocomial infections including UTIs and account for 15%-30% of CAUTIs (8). Enterococcus faecalis isolates can produce biofilms on urinary catheters and grow despite an intense inflammatory response (9,10).

In this context, this study was divided in two parts. In the first part, we aimed to evaluate the ability of biofilm formation in *E. faecalis* clinical isolates from urine infections. In the second part, we aimed to evaluate the antimicrobial durability and the inhibition of biofilm formation in different types of commercial urinary catheters.

## MATERIALS AND METHODS

This study was approved by the ethics committee.

### Strains used in the study

A total number of 89 *E. faecalis* clinical isolates were included in this study. These isolates were taken from urinary catheter (n=45) and urine samples (n=44) of hospitalized intensive care unit patients (n=19 internal medicine, n=25 neurosurgery, n=14 general surgery, n=15 neurology, n=16 emergency room) who were admitted to a University Hospital from 2000 to 2011.

In addition to *E. faecalis*, isolates of 45 hospitalized patients with catheters and laboratory strains of E. coli ATCC25922, *E. faecalis* ATCC29212, *P. aeruginosa* ATCC27853, S. epidermidis ATCC35984 and *C. albicans* ATCC90028 were used in catheter assays.

### Microtiter plate assays

**Biofilm formation and quantification:** Final inoculum suspensions of all clinical *E. faecalis* strains were adjusted to approximately 106 colony forming units (CFU) mL-1. Each experiment included the biofilm-forming *E. faecalis* ATCC29212 strain as a positive control. Sterile tryptic soy broth (TSB) (Becton Dickinson GmbH, Heidelberg, Germany) with 0.25% glucose was used as the blank.

For each test condition, 12 wells of a flat-bottomed polystyrene 96-well microtiter plate were inoculated with 100 μL of the final inoculum suspension. After 4 h of incubation at 37 °C without shaking, non-adhered cells were removed and rinsed with 100 μL of 0.9% physiological saline (PS), then 100 μL of fresh TSB with 0.25% glucose was added, and the plates were incubated for an additional 20 h for biofilm maturation. After 24 h, supernatants were removed, and each well was rinsed with PS before quantifying the sessile cells.

**Crystal violet staining:** The biomass quantification of *E. faecalis* biofilms was performed according to an optimized assay (11). After rinsing of the wells, 100 μL of a solution of 0.2% crystal violet (CV) was added for 15 min, the stained biofilms were rinsed again to remove excess dye and dried for 15 min at room temperature. The bound dye was solubilized using acetone/ethanol. The optical densities (ODs) of the stained adherent bacterial cells were measured at 570 nm using a micro-ELISA plate reader. We defined the cut-off OD (0.282) as three standard deviations above the mean OD of the negative control. Each isolate was tested in twelve wells in each assay, and each assay was carried out in duplicate (n=24).

### Plate counting

The number of cells in mature biofilms was quantified via plate counting using tryptic soy agar (TSA) medium. Biofilms were detached by vortexing (5 min) followed by sonication (5 min). Serial dilutions of sonication fluids were made and plated on TSA to determine the number of (CFU) per millilitre of the isolates. Each isolate was tested in twelve wells in each assay, and each assay was carried out in duplicate (n=24).

### Catheter assays

### Foley catheters used in the study

We tested five types of commercially 18 Fr Foley catheters including:

1. A nitrofurazone-impregnated silicone catheter (RelaseNF®),

2. A silver-coated silicone catheter (Dover®),

3. A hydrophilic-coated silicone catheter without an antimicrobial agent,

4. A silico-latex catheter (Rüsch®) without antimicrobial agent, and

5. A silicone catheter without an antimicrobial agent (Rüsch®).

Catheters were cut into 1-cm long segments followed by cutting in half lengthwise to expose the interior surface.

### Antimicrobial durability in catheters

The antimicrobial durability of the nitrofurazone-impregnated and the silver-coated silicone catheter was assessed over time. We used a previously described method, with some modifications (12). Briefly, sterile catheter segments were placed in sterile 50-mL Falcon polystyrene tubes containing 10 mL of sterile Mueller-Hinton broth (MHB) (Becton Dickinson GmbH, Heidelberg, Germany) and incubated at 37 °C for 0, 1, 3, and 5 days in duplicate. MHB medium was changed at daily intervals. After the pre-incubation period, the segments were placed on MHB agar, and zones of inhibition were determined using the modified Kirby-Bauer method against E. coli ATCC25922, *E. faecalis* ATCC29212, S. epidermidis ATCC35984, *P. aeruginosa* ATCC27853 and *C. albicans* ATCC90028. Each catheter was tested in duplicate in each assay.

### Biofilm formation in catheters

Sterile catheter segments were placed in sterile 24-well tissue culture plates (Corning Costar, Corning, NY, USA) and 1 mL of TSB containing approximately 106 CFU mL-1 of the 45 *E. faecalis* isolates obtained from the inpatients with catheters, E. coli ATCC25922, *E. faecalis* ATCC29212, *P. aeruginosa* ATCC27853 and S. epidermidis ATCC35984 and approximately 105 CFU mL-1 of *C. albicans* ATCC90028 were added separately to the wells for each catheter. The plates were then placed individually in the incubator at 37 °C for 24 hours for biofilm formation. After the incubation time, the contents of the wells were removed and segments were washed three times in 0.9% PS and aseptically transferred to tubes containing 1 mL phosphate-buffered saline. Then, the tubes were sonicated (15 min) and vortexed (30 seconds) three times. Serial dilutions were made in sterile PS and plated onto TSA. Each catheter was tested three times in each assay.

### Scanning electron microscopy in catheters

The catheter segments were washed with sterile distilled water following 24 hours of incubation. Then, the segments were fixed in a buffer containing 2% glutaraldehyde and 0.1 M cacodylate for 30 min followed by rinsing three times for 10 min in 0.2 M cacodylate buffer. After passage through serial ethanol solutions, samples were dried and then coated with gold-palladium (13).

### Statistical analysis

Statistical analysis was performed using the software program Statistical Package Version 21 (SPSS Inc.; Chicago, IL, USA). The normal or non-normal distributions of the data were verified using the Shapiro-Wilk test. For the comparison of urinary catheters, we used the one-way ANOVA test.

## RESULTS

### Detection of biofilm production by *E. faecalis* isolates

**FIG. 1.**
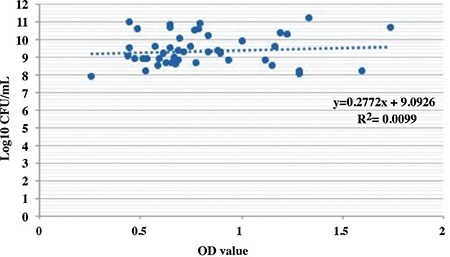
Biofilm forming ability of *E. faecalis* isolates from the urinary catheter samples of inpatients.

**FIG. 2.**
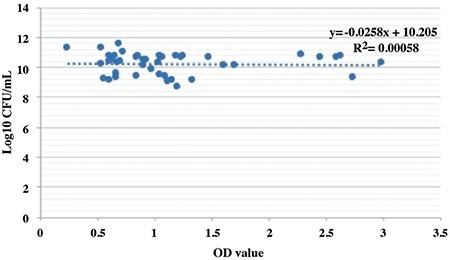
Biofilm forming ability of *E. faecalis* isolates from the urine samples of hospitalized patients without catheters.

In this study, 89 *E. faecalis* isolates were tested to assess biofilm formation ability by two different methods. All isolates produced biofilms as determined by the plate counting assay, and in terms of biofilm forming ability, no statistically significant difference was determined between the isolates from the inpatients with and without catheters. Although all isolates from the inpatients with catheters were detected as biofilm positive by the CV staining assay, only 2 of 44 isolates from the urine samples of the inpatients were found to be biofilm negative. The results obtained by the CV staining assay and plate counting method for the isolates from the inpatients with and without catheters are presented in Figures 1 and 2, respectively.

### Antimicrobial durability of urinary catheters

The antimicrobial effects of the nitrofurazone-impregnated silicone catheter were observed immediately after the start of incubation and at the end of the first and fifth days against *C. albicans*, *E. faecalis* and S. epidermidis, and E. coli and *P. aeruginosa*, respectively. This catheter was found to be more effective against gram-negative than gram-positive bacteria and to have no effect on *C. albicans*, as no inhibition zone was detected. However, for the second tested silver-coated silicone catheter, an inhibition zone was not detected just after the incubation period (data not shown).

### Determination of the effects of various urinary catheters on biofilms formed by clinical isolates and laboratory strains

**FIG. 3.**
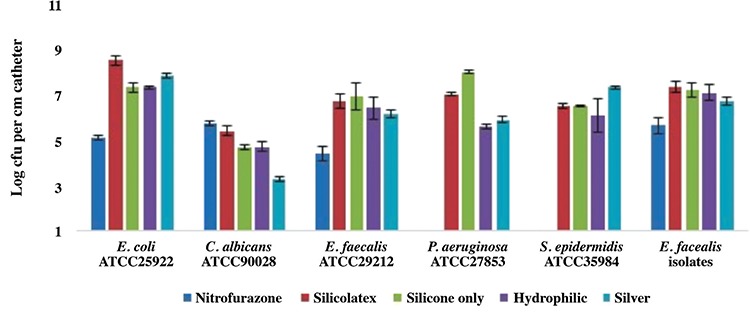
Effects of urinary catheters on biofilms formed by laboratory strains and 45 *E. faecalis* clinical isolates. Results are means of three different experiments. The mean log CFU/mL of biofilm cells on nitrofurazone impregnated catheters was significantly less than on the others, except in the case of *C. albicans* ATCC-90028 (p<0.001). For the *C. albicans* strain, the mean log CFU/mL of biofilm cells on the silver-coated catheter was significantly less than on the others.

We compared different urinary catheters to determine their effects on biofilms of clinical isolates and laboratory strains. Figure 3 shows the mean log10 CFU/mL of the biofilm cells of 45 *E. faecalis* isolates obtained from the hospitalized patients with catheters and laboratory strains per centimetre of catheter segment after 24 hours of incubation. The number of viable biofilm cells of the clinical isolates on the nitrofurazone-impregnated silicone catheter was lower than that on the silver- or hydrophilic- coated catheters, and the difference between catheters was found to be statistically significant (p<0.001) (Figure 3).

Among the laboratory strains of E. coli, *E. faecalis*, *P. aeruginosa*, S. epidermidis and *C. albicans*, the nitrofurazone-impregnated catheter completely inhibited the growth of viable cells of *P. aeruginosa* and S. epidermidis after 24 hrs of incubation. The mean log10 counts of viable sessile cells on the nitrofurazone-impregnated silicone catheter, except for those of *C. albicans*, was significantly lower than on the silver- and hydrophilic-coated silicone catheters (p<0.05) (Figure 3).

### Biofilm characterization by scanning electron microscopy

**FIG. 4.**
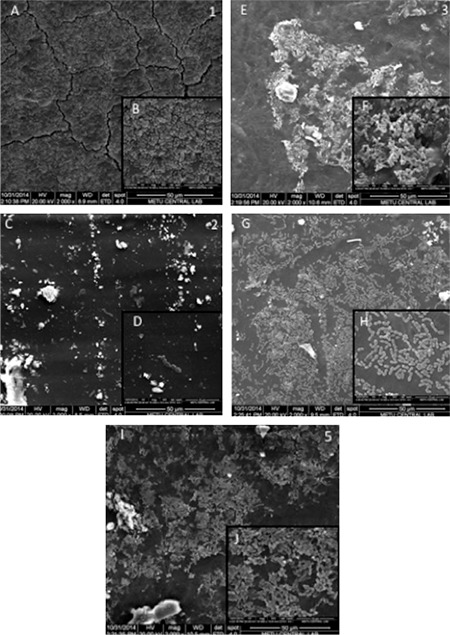
Scanning electron micrographs of the *E. faecalis* ATCC 29212 biofilms formed in the urinary catheters. 1. Silico-latex catheter 2. Nitrofurazone-impregnated silicone catheter 3. Hydrophilic silicone catheter 4. Silver-coated silicone catheter 5. Silicone only catheter A: SEM images magnified x2000, B: SEM images magnified x10.000.

The scanning electron microscope (SEM) results for *E. faecalis* ATCC 29212 were consistent with the results of the plate counting method. As in Figure 4, there were fewer sessile cells on the nitrofurazone-impregnated catheter compared with the others. The silver-coated urinary catheter was the second most effective catheter, showing a reduced number of sessile cells of *E. faecalis* (Figure 4).

## DISCUSSION

Biofilm formation on the surface of Foley catheters is the major cause of bacteriuria. The biofilm layer formed by bacteria attached to the urinary catheter surface is the major challenging problem for treatment of CAUTIs (6,14,15). In our study, the biofilm forming ability of *E. faecalis* isolates in microtiter plates was analysed both by CV staining and plate counting assay. When we compared the isolates from inpatients with and without urinary catheters, the results indicate that all isolates were biofilm positive by the plate counting method. For all samples, although the starting bacteria concentration was normalized to 6 log10 (106 CFU/mL) after the incubation period, the lowest bacterial number in the wells was found to be 7.8 log10, indicating that all the isolates attached and grew on the walls of the wells in microtiter plates (Figure 1). In plate counting assay, all isolates (both for hospitalized patients with and without urinary catheters) were found to be biofilm positive. However, two of the isolates from the urine samples of the hospitalized patients without catheters which were found to be biofilm positive in the plate counting assay (log10 CFU/mL results were 10.2 and 11.1) were detected as biofilm negative in the CV staining assay (Figure 2). This minor difference in our results may be due to the separate targets of each method for the quantification of biofilm cells. Although minor discrepancies in the results in terms of biofilm formation ability was found using these two methods, our results reveal that a high percentage of *E. faecalis* isolates from inpatients produced a significant amount of biofilm with these two methods.

The CV dye used in the CV staining assay stains both living and dead cells by linking to negatively charged surface molecules and polysaccharides in the ECM. It was shown in previous studies that the degree of exopolysaccharide matrix content in the biofilm formed by an isolate could influence the biofilm formation results when comparing the CV staining assay based on biomass quantification and the plate counting assay based on cell viability (12). ECM content and structure may vary based on the cell type and experimental design; therefore, no correlation between CFU and OD values in CV staining was expected. As shown in Figures 1 and 2, no correlation between the CV staining and plate counting assay was determined by regression analysis. Consequently, with this analysis our hypothesis concerning two methods having different targets to quantify the biofilm cells was supported. Although the CV staining method is used as the gold standard for the in vitro determination of biofilm formation to quantify biofilm biomass, there are many studies using variable conditions such as the number of washing steps, the conditions of washing, the duration of CV staining, the concentration of CV used as well as different cut-off OD values, etc. to classify isolates with this method (16,17,18,19). For these reasons, results regarding the identification of the biofilm forming ability of isolates also vary widely among studies.

The occurrence of CAUTIs, as the most common hospital acquired infection, has an important economic and clinical impact and is directly related to the majority of uropathogens able to form biofilms, including E. coli, *E. faecalis*, *P. aeruginosa*, S. aureus, P. mirabilis and *C. albicans*. *E. faecalis* is an opportunistic pathogen and is also considered a significant uropathogen in CAUTI, since catheterization can have a negative effect on the defences of the patient against this pathogen. It is widely known that the presence of bacterial biofilms on the inner or outer surface of the catheter leads to CAUTI (10). Researchers reported that the novel antimicrobial Foley catheter coatings had a potential in the prophylaxis of catheter-related UTIs and reduced biofilm formation (20,21).

The present study compared the effects of different urinary catheters on inhibiting biofilm formations of *E. faecalis* isolates obtained from the urinary catheters of 45 inpatients and laboratory strains in vitro. Viable sessile cell counts of *E. faecalis* isolates were the lowest for the nitrofurazone-impregnated silicone catheter of all the catheters tested (p<0.001). Mean sessile cell numbers of *E. faecalis* isolates on the silver-coated catheter were significantly lower than on the silicolatex and silicone catheters (p<0.001, p=0.005, respectively); however, there was no significant difference between the silver- and hydrophilic-coated catheters, p=0.286). Our results reveal that the nitrofurazone-impregnated silicone catheter is the most effective at reducing the number of sessile cells of *E. faecalis* isolates. These findings agree with previous in vitro assessments of antimicrobial catheters (22). It was concluded that the adherence of *E. faecalis* to the nitrofurazone-impregnated and hydrogel-coated silicone catheters was significantly decreased compared with the silicone-only catheters. Furthermore, the silver coating had little effect on bacterial adherence, whereas nitrofurazone impregnation had a significant effect that lasted up to 5 days (8).

Our data also reveal that the nitrofurazone-impregnated silicone catheter showed more prolonged antimicrobial durability when compared with the silver-coated catheter against E. coli, *P. aeruginosa*, S. epidermidis and *E. faecalis* laboratory strains. It was shown in a clinical trial study that nitrofurazone-impregnated catheters are beneficial in reducing asymptomatic bacteriuria with <1 week of catheterization (7). The present study shows that the antimicrobial effect of the nitrofurazone-impregnated catheter decreased over time, and the antimicrobial durability showed variability depending on the strain tested in this study (data not shown). Previous studies have also consistently shown decreased inhibition by the nitrofurazone-coated catheter over time. However, the decline in the duration of antimicrobial activity obtained from our study at 1 day for *E. faecalis* and S. epidermidis was more rapid than that observed in some previous studies (23). This difference may have arisen from the different conditions in vitro and in vivo.

The nitrofurazone-impregnated silicone catheter fully inhibited the growth of *P. aeruginosa* and S. epidermidis biofilms. For *P. aeruginosa*, the hydrophilic-coated catheter was significantly more effective than the silico-latex and silicone only catheters, while for S. epidermidis, the silver-coated catheter was significantly more effective than the hydrophilic-coated catheter (p<0.001). The number of viable biofilm cells of E. coli and *E. faecalis* on the nitrofurazone-impregnated silicone catheter was significantly lower than that on the silver-coated silicone, hydrophilic-coated silicone, silicone-only and silico-latex catheters (p<0.001) (Figure 3). For *C. albicans*, the silver-coated silicone catheter was the most effective at reducing the number of sessile cells. In a comparative multicentre study, Enterococcus species were found to be the most prevalent gram-positive bacteria identified in CAUTI; the incidence rate of CAUTI was lower in patients who were catheterized for 5-7 days and in older patients using a nitrofurazone-coated catheter compared with silicone only catheters (24). Another study reported that nitrofurazone-coated catheters insistently prevented the growth of the causative organisms of CAUTI for 2-5 days, whereas silver hydrogel catheters only inhibited the growth of Staphylococcus species for less than 1 day in vitro (25).

SEM analysis revealed an intense network of cellular layers consistent with the results of sessile cell counts of *E. faecalis* obtained from urinary catheters. The nitrofurazone-impregnated catheter was found to be the catheter with the lowest number of *E. faecalis* cells, followed by the silver-coated urinary catheter.
